# Mesenchymal stem cell-derived exosomes promote scalp rejuvenation through type XVII collagen regulation via the miR-21-5p/DKK2/Wnt pathway

**DOI:** 10.3389/fcell.2026.1807877

**Published:** 2026-04-22

**Authors:** Shiyue Cui, Yuanyuan Huang, Duo Zeng, Jie Li, Chun Tang

**Affiliations:** 1 Bravou Medical Aesthetic Hospital, Chengdu, China; 2 Wuzhou Plastic and Cosmetic Surgery Hospital, Wuhan, China; 3 Changsha Yamei Medical Aesthetic Plastic Surgery Hospital, Changsha, China; 4 Kunming Han Chen Medical Aesthetic Hospital, Kunming, China

**Keywords:** COL17A1, DKK2, exosomes, hair follicle stem cells, mesenchymal stem cells, MiR-21-5p, Wnt/β-catenin

## Abstract

**Background:**

Hair follicle aging is driven by COL17A1 proteolysis in hair follicle stem cells (HFSCs). Mesenchymal stem cell (MSC)-derived exosomes show promise in tissue regeneration, but their effects on COL17A1 and hair follicle aging remain unexplored.

**Methods:**

Exosomes isolated from human umbilical cord MSCs (hUC-MSCs) were evaluated in H_2_O_2_-induced senescent human dermal papilla cells (hDPCs), *ex vivo* human hair follicle organ culture, and C57BL/6 mouse hair regeneration model. Small RNA sequencing, dual-luciferase reporter assay, and rescue experiments were performed to elucidate the mechanism.

**Results:**

Exosome treatment reduced SA-β-gal-positive cells from 67.5% to 21.4%, upregulated COL17A1 mRNA by 2.67-fold (with protein restoration to 0.89-fold of control levels), enhanced hair follicle elongation by 47.4%, and achieved 92.4% hair coverage in mice versus 45.6% for controls, outperforming minoxidil (78.3%). miR-21-5p was identified as the most abundant exosomal miRNA and confirmed to directly target DKK2, a Wnt antagonist. DKK2 suppression was associated with Wnt/β-catenin pathway activation and upregulation of COL17A1 expression. miR-21-5p inhibitor partially blocked exosome effects, validating this axis.

**Conclusion:**

hUC-MSC-derived exosomes attenuate hair follicle senescence and promote hair regeneration through the miR-21-5p/DKK2/Wnt/β-catenin/COL17A1 axis, providing a promising cell-free strategy for scalp rejuvenation.

## 1 Introduction

Scalp aging is a universal physiological process characterized by progressive hair follicle miniaturization, hair shaft thinning, decreased hair density, and eventual hair loss (Jang et al., 2023). These changes significantly impact quality of life and psychological wellbeing, driving a growing demand for effective anti-aging interventions. Hair follicle stem cells (HFSCs), residing in the bulge region, are essential for hair cycling and regeneration throughout life (Zhang et al., 2021; Matsumura et al., 2021). These stem cells undergo repeated cycles of activation (anagen), regression (catagen), and quiescence (telogen) to sustain hair production. However, with advancing age, HFSCs progressively lose their regenerative capacity, leading to hair follicle miniaturization and hair loss (Shin et al., 2020). While various treatments including minoxidil, finasteride, and hair transplantation are available, their efficacy remains limited, and the fundamental mechanisms underlying hair follicle aging are not fully addressed by current therapeutic approaches (Kaiser et al., 2023; Gupta et al., 2022).

Type XVII collagen (COL17A1, also known as BP180) has emerged as a central regulator of HFSC fate and hair follicle homeostasis (Matsumura et al., 2016; Watanabe et al., 2017). COL17A1 is a transmembrane protein that anchors HFSCs to the basement membrane through hemidesmosome complexes. The landmark study by Matsumura et al. published in Science (2016) revealed that COL17A1 proteolysis is the key trigger for HFSC aging. They demonstrated that aged HFSCs exhibit reduced COL17A1 expression, leading to destabilization of hemidesmosomes, loss of stem cell anchorage, aberrant differentiation, and eventual elimination through transepidermal shedding. This seminal finding established COL17A1 as a master gatekeeper of HFSC stemness and a promising therapeutic target for hair follicle anti-aging interventions (Matsumura et al., 2021; Han et al., 2022).

Mesenchymal stem cell (MSC)-derived exosomes have attracted considerable attention as novel therapeutic agents for tissue regeneration (Zhou et al., 2024; Cheng et al., 2024; Kost et al., 2022). Exosomes are nanoscale extracellular vesicles (30–150 nm) carrying bioactive cargo including proteins, lipids, and microRNAs (miRNAs) that modulate recipient cell function (Théry et al., 2018). Compared to cell-based therapies, exosomes offer advantages of low immunogenicity, no tumorigenicity, and standardized manufacturing (Bai et al., 2024). Recent studies have explored exosomes in hair regeneration: Chen et al. (2020) reported that dermal papilla cell-derived exosomes promote hair growth through miR-140-5p-mediated BMP signaling modulation, and Kim et al. (2022) demonstrated that colostrum-derived exosomes accelerate hair regeneration via Wnt/β-catenin pathway activation. However, whether exosomes can specifically target COL17A1 to counteract HFSC aging remains unexplored (Hu et al., 2020; Wu et al., 2021; Lee E. et al., 2024).

Based on these observations, we hypothesized that MSC-derived exosomes may attenuate HFSC senescence by delivering specific miRNAs that activate the Wnt/β-catenin pathway, thereby upregulating COL17A1 expression. The Wnt/β-catenin pathway is a master regulator of hair follicle cycling (Liu J. et al., 2022; Choi, 2020), and DKK family members (including DKK1 and DKK2) are secreted Wnt antagonists associated with hair follicle regression (Song et al., 2018; Lee S. B. et al., 2024). In this study, we aimed to: (1) investigate the effects of human umbilical cord MSC-derived exosomes on hair follicle-associated cellular senescence and COL17A1 expression, using dermal papilla cells as the primary *in vitro* model given their central role as paracrine signaling hubs that regulate hair follicle stem cell niche maintenance; (2) elucidate the molecular mechanism by which exosomes regulate COL17A1; and (3) evaluate the therapeutic potential of exosomes for scalp rejuvenation and hair regeneration.

## 2 Materials and methods

### 2.1 Ethics statement

The studies involving human participants were reviewed and approved by the Institutional Review Board of Bravou Plastic Surgery Hospital, Chengdu, China (Approval No.: LLDWYH-2024001, Valid: 30 January 2024 to 30 January 2026). All procedures were performed in accordance with the Declaration of Helsinki. Written informed consent was obtained from all participants prior to tissue collection. The animal study was conducted following the ARRIVE guidelines and NIH Guide for the Care and Use of Laboratory Animals.

Inclusion criteria required that participants had discontinued all pharmacological treatments for alopecia (including topical minoxidil and oral finasteride) for at least 3 months prior to tissue collection.

### 2.2 Human samples and cell culture

#### 2.2.1 Human scalp tissue collection

Human scalp tissue samples were obtained from the occipital donor area of patients undergoing hair transplantation surgery. The discarded tissue was collected for research purposes. Inclusion criteria were: (1) age 25–55 years; (2) generally healthy; (3) provided written informed consent. Exclusion criteria were: (1) autoimmune diseases; (2) use of hair growth medications within 3 months; (3) chronic systemic diseases; (4) scalp infections. A total of 25 donors were enrolled. Fresh samples were transported to the laboratory in ice-cold PBS within 2 h and coded for de-identification.

#### 2.2.2 Cell culture

Human umbilical cord mesenchymal stem cells (hUC-MSCs) were purchased from Cyagen Biosciences (Guangzhou, China; Cat. No. HUXUC-01001) and cultured in α-MEM supplemented with 10% FBS and 1% penicillin-streptomycin at 37 °C with 5% CO_2_. Cells at passages 3–5 were used for exosome isolation. Cell identity was verified by flow cytometry (positive: CD73, CD90, CD105; negative: CD34, CD45).

Human dermal papilla cells (hDPCs) were selected as the primary *in vitro* model because they function as key paracrine signaling hubs that regulate hair follicle cycling and HFSC niche homeostasis through secretion of growth factors including BMP, FGF, and Wnt ligands, and because they represent the direct recipient cell population for exosome cargo delivered to the scalp microenvironment. hDPCs were isolated from scalp tissue by microdissection. The dermal papilla was dissected under a stereomicroscope and digested with 0.2% type I collagenase (Sigma-Aldrich) at 37 °C for 1 h hDPCs were cultured in DMEM supplemented with 10% FBS and 10 ng/mL bFGF (PeproTech). Cells at passages 4–8 were used for experiments.

### 2.3 Exosome isolation and characterization

Exosomes were isolated from hUC-MSC conditioned medium by differential ultracentrifugation. Cells were cultured in serum-free medium for 48 h. The conditioned medium was sequentially centrifuged at 300 *g* (10 min), 2,000 *g* (30 min), and 10,000 *g* (45 min) to remove cells and debris, filtered through 0.45 μm filters, and ultracentrifuged at 100,000 *g* for 70 min (Beckman SW 32 Ti rotor). The pellet was washed with PBS and resuspended for use.

Exosomes were characterized according to MISEV2018 guidelines (Théry et al., 2018). Morphology was examined by transmission electron microscopy (TEM). Size distribution and particle concentration were analyzed by nanoparticle tracking analysis (NTA, NanoSight NS300). Protein concentration of the exosome preparation was quantified by BCA assay, yielding a protein output of 124 ± 18 µg per mL of conditioned medium and a particle-to-protein ratio of 2.6 × 10^8^ particles/µg, consistent with values reported for high-purity MSC-derived exosome preparations. Protein markers were verified by Western blot (positive: CD9, CD63, CD81, TSG101; negative: Calnexin). Antibody information is provided in Supplementary Table S1. To confirm intraluminal encapsulation of miR-21-5p, an RNase A protection assay was performed. Exosome preparations were treated with RNase A (1 μg/mL, 37 °C, 30 min) in the presence or absence of 0.1% Triton X-100, followed by miR-21-5p quantification by qRT-PCR. miR-21-5p levels were largely preserved following RNase A treatment alone (85.3% ± 6.2% of untreated control), but were markedly reduced upon co-treatment with Triton X-100 (8.7% ± 2.1%), confirming that miR-21-5p is encapsulated within the exosomal lumen rather than surface-associated (Supplementary Figure S1).

For uptake assay, exosomes were labeled with PKH26 red fluorescent dye (Sigma-Aldrich), incubated with hDPCs for 0, 6, 12, and 24 h, and visualized by confocal microscopy. We acknowledge that PKH26 labeling is associated with known methodological limitations, including the potential formation of dye micelles that may co-pellet with exosomes during ultracentrifugation and the persistence of membrane-incorporated dye independently of the exosome vehicle. These factors may introduce artifacts in uptake quantification. Future studies employing alternative labeling strategies, such as CD63-GFP-expressing donor cells or lipophilic carbocyanine dyes with shorter membrane retention, would provide more rigorous confirmation of exosome internalization.

### 2.4 *In vitro* and ex vivo experiments

#### 2.4.1 *In vitro* experiments

hDPCs senescence was induced by 200 μM H_2_O_2_ treatment for 2 h hDPCs were isolated from 6 independent donors and pooled in pairs for experiments. Cells were divided into five groups, and all experiments were performed as three independent replicates (n = 3), each using hDPCs pooled from 2 donors: (1) Control; (2) Model (H_2_O_2_ only); (3) Exo-L (H_2_O_2_ + 25 μg/mL exosomes, corresponding to approximately 6.5 × 10^9^ particles/mL); (4) Exo-M (H_2_O_2_ + 50 μg/mL, approximately 1.3 × 10^10^ particles/mL); (5) Exo-H (H_2_O_2_ + 100 μg/mL, approximately 2.6 × 10^10^ particles/mL). Particle concentrations were calculated based on the particle-to-protein ratio determined by NTA and BCA assay (2.6 × 10^8^ particles/µg). Exosome treatment was maintained for 48 h.

Cell proliferation was assessed by CCK-8 assay (Dojindo) at 24, 48, and 72 h. Cellular senescence was evaluated using SA-β-galactosidase Staining Kit (Cell Signaling Technology) at pH 6.0. For Wnt pathway inhibition experiments, cells were pretreated with 10 μM IWR-1 (Sigma-Aldrich) for 2 h before exosome treatment.

#### 2.4.2 *Ex vivo* hair follicle organ culture

Intact anagen hair follicles were isolated by microdissection and cultured individually in Williams’ E medium supplemented with 10 ng/mL hydrocortisone, 10 μg/mL insulin, and 1% penicillin-streptomycin. Follicles were randomly assigned to PBS control (n = 15) or exosome treatment (100 μg/mL, n = 15) groups. Medium was replaced every other day for 9 days. Hair follicle length was measured daily from the dermal papilla base to the hair shaft tip. At the endpoint, follicles were processed for histological analysis (Langan et al., 2015).

### 2.5 *In vivo* animal experiments

Seven-week-old male C57BL/6J mice (n = 32) were housed under SPF conditions. Dorsal hair was removed to synchronize follicles in telogen phase. Mice were randomly divided into four groups (n = 8 per group): (1) PBS (intradermal injection); (2) Minoxidil (2.5%, topical, daily); (3) Exo-L (50 μg, intradermal); (4) Exo-H (100 μg, intradermal). Intradermal injections (100 μL total, 5 sites × 20 μL) were administered every other day for 21 days.

Hair regeneration was photographed and quantified (hair coverage area) at days 0, 7, 14, and 21. Image analysis was performed by two investigators blinded to group allocation using ImageJ software, and the results were averaged to minimize observer bias. At day 21, mice were euthanized by CO_2_ inhalation and dorsal skin was collected for histological and molecular analyses.

### 2.6 Molecular and histological analyses

Western blot: Proteins were extracted using RIPA buffer with protease/phosphatase inhibitors, separated by SDS-PAGE, transferred to PVDF membranes, and probed with primary antibodies overnight at 4 °C. Antibody details are listed in Supplementary Table S1. Bands were visualized by ECL and quantified using ImageJ.

qRT-PCR: Total RNA was extracted using TRIzol (Invitrogen) and reverse-transcribed using PrimeScript RT Kit (TaKaRa). qPCR was performed using TB Green Premix (TaKaRa). Primer sequences are listed in Supplementary Table S2. Relative expression was calculated by 2^(-ΔΔCt) method with GAPDH as internal control. For miRNA quantification, U6 served as control.

Immunofluorescence and immunohistochemistry: For immunofluorescence, cells were fixed with 4% paraformaldehyde, permeabilized, blocked, incubated with primary antibodies, and visualized with Alexa Fluor-conjugated secondary antibodies. Nuclei were stained with DAPI. For IHC, paraffin sections underwent antigen retrieval and DAB chromogen development. Staining was quantified using Image-Pro Plus.

H&E staining: Tissues were fixed in 10% formalin, paraffin-embedded, sectioned (5 μm), and stained with hematoxylin and eosin for morphological analysis.

Small RNA sequencing and bioinformatics: Exosomal RNA was extracted using miRNeasy Mini Kit (Qiagen), and libraries were sequenced on Illumina NovaSeq 6000. Reads were aligned to miRBase v22.1. Target genes were predicted using TargetScan and miRDB. GO/KEGG enrichment analyses were performed using clusterProfiler.

Dual-luciferase reporter assay: The DKK2 3′UTR containing the miR-21-5p binding site (wild-type or mutant) was cloned into pmirGLO vector. HEK293T cells were co-transfected with reporter plasmid and miRNA mimic using Lipofectamine 3000. Luciferase activity was measured at 48 h using Dual-Luciferase Reporter System (Promega).

Transfection: hDPCs were transfected with miRNA mimics or inhibitors (GenePharma) using Lipofectamine RNAiMAX. For rescue experiments, inhibitor-transfected cells were treated with exosomes 6 h post-transfection.

TOP/FOP Flash assay: hDPCs were transfected with TOPflash or FOPflash reporter plasmid and pRL-TK control. After exosome treatment, Wnt pathway activity was expressed as the TOPflash/FOPflash ratio.

### 2.7 Statistical analysis

Statistical analyses were performed using GraphPad Prism 9.0. Data are presented as mean ± SD from at least three independent experiments. Prior to parametric testing, normality of all datasets was confirmed using the Shapiro-Wilk test. Comparisons between two groups were analyzed by unpaired Student’s t-test. Comparisons among multiple groups were analyzed by one-way ANOVA followed by Tukey’s *post hoc* test. Two-way ANOVA was used for time-course experiments. For *ex vivo* hair follicle elongation data, a linear mixed-effects model with donor as a random effect was applied to account for the correlation among follicles derived from the same donor. Post-hoc power analysis for the two primary endpoints (hair coverage percentage and SA-β-gal-positive cell ratio) confirmed adequate statistical power (β > 0.80) for the sample sizes employed. P < 0.05 was considered statistically significant (*P < 0.05, **P < 0.01, ***P < 0.001). Raw data distribution plots for all quantitative analyses are provided in Supplementary Figure S2.

## 3 Results

### 3.1 Characterization of hUC-MSC-derived exosomes and cellular uptake

**FIGURE 1:**
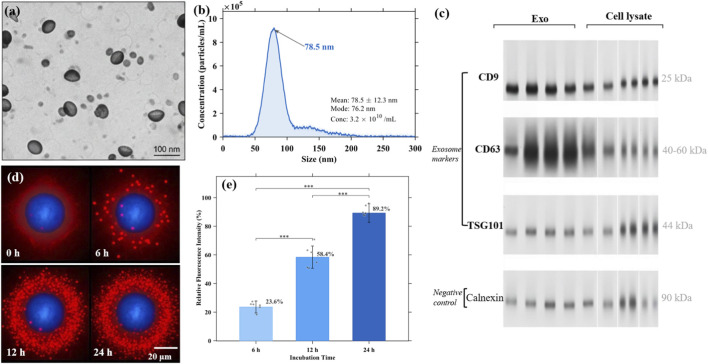
Characterization of hUC-MSC-derived exosomes and cellular uptake by hDPCs. **(a)** TEM. Scale bar = 100 nm. **(b)** NTA size distribution. **(c)** Western blot of exosome markers. **(d)** Confocal imaging of PKH26-labeled exosome uptake at 0–24 h. Red, PKH26; blue, DAPI. Scale bar = 20 μm. **(e)** Uptake quantification.

To obtain high-quality exosomes for subsequent experiments, we isolated exosomes from hUC-MSC conditioned medium by differential ultracentrifugation and characterized them according to MISEV2018 guidelines (Théry et al., 2018). Transmission electron microscopy (TEM) revealed that the isolated particles exhibited typical cup-shaped or saucer-like morphology characteristic of exosomes, with diameters ranging from 40 to 120 nm (Figure 1a). Nanoparticle tracking analysis (NTA) showed that the particle size distribution ranged from 30 to 150 nm, with a peak at 78.5 ± 12.3 nm, and the concentration was approximately 3.2 × 10^10^ particles/mL (Figure 1b). Western blot analysis confirmed that the isolated exosomes were positive for tetraspanin markers CD9, CD63, and CD81, as well as ESCRT-associated protein TSG101, while negative for the endoplasmic reticulum marker Calnexin (Figure 1c), indicating successful isolation of exosomes without cellular contamination.

To verify whether exosomes could be internalized by target cells, we labeled exosomes with PKH26 red fluorescent dye and incubated them with hDPCs for various time periods. Confocal microscopy revealed that PKH26-labeled exosomes were effectively taken up by hDPCs, with red fluorescence signals distributed mainly in the perinuclear cytoplasm (Figure 1d). Quantitative analysis showed that exosome uptake increased in a time-dependent manner, with fluorescence intensity reaching 23.6% ± 4.2%, 58.4% ± 7.8%, and 89.2% ± 6.5% at 6, 12, and 24 h, respectively, compared to the 24-h maximum (Figure 1e).

To confirm that miR-21-5p is encapsulated within the exosomal lumen rather than surface-associated or present as free RNA in the preparation, an RNase A protection assay was performed. miR-21-5p levels were largely preserved following RNase A treatment alone (85.3% ± 6.2% of untreated control), but were markedly reduced upon co-treatment with Triton X-100 (8.7% ± 2.1%), confirming intraluminal encapsulation (Supplementary Figure S1).

### 3.2 Exosomes attenuate H_2_O_2_-Induced senescence and upregulate COL17A1 in hDPCs

A total of 25 donors (18 males and 7 females, mean age 38.5 ± 8.2 years, range 25–55 years) were enrolled in this study. The demographic characteristics of scalp tissue donors are summarized in Table 1.

**TABLE 1:** Characteristics of human scalp tissue donors (n = 25).

Characteristic	Value
Age (years)	​
- Range	25–55
- Mean ± SD	38.5 ± 8.2
- 25–35 years, n (%)	8 (32.0%)
- 36–45 years, n (%)	10 (40.0%)
- 46–55 years, n (%)	7 (28.0%)
Sex, n (%)	​
- Male	18 (72.0%)
- Female	7 (28.0%)
Ethnicity, n (%)	​
- Chinese (East Asian)	25 (100%)
Alopecia classification, n (%)	​
- Males: Hamilton-Norwood (n = 18)	​
Grade II	3 (16.7%)
Grade III	5 (27.8%)
Grade III vertex	4 (22.2%)
Grade IV	4 (22.2%)
Grade V	2 (11.1%)
- Females: Ludwig (n = 7)	​
Grade I	3 (42.9%)
Grade II	3 (42.9%)
Grade III	1 (14.3%)
Alopecia duration (years)	​
- Range	2–15
- Mean ± SD	6.8 ± 3.5
Prior treatment history (discontinued ≥3 months), n (%)	​
- Topical minoxidil	14 (56.0%)
- Oral finasteride (males, n = 18)	8 (44.4%)
- No prior pharmacotherapy	6 (24.0%)
Surgery type, n (%)	​
- FUT (follicular unit transplantation)	15 (60.0%)
- FUE (follicular unit extraction)	10 (40.0%)
Sample allocation, n	​
- Primary cell isolation	10
- Hair follicle organ culture	10
- Direct tissue analysis	5

Categories for prior treatment history are not mutually exclusive; some patients received both topical minoxidil and oral finasteride.

To investigate the anti-senescence effects of exosomes, we established an H_2_O_2_-induced premature senescence model in hDPCs and treated cells with different concentrations of exosomes for 48 h.

**FIGURE 2:**
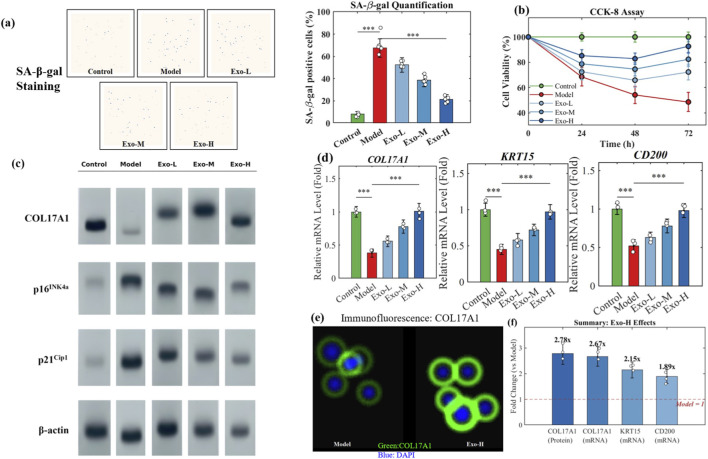
Exosomes attenuate H_2_O_2_-induced senescence and upregulate COL17A1. **(a)** SA-β-gal staining and quantification. **(b)** CCK-8 assay. **(c)** Western blot of COL17A1, p16, and p21. **(d)** qRT-PCR. **(e)** COL17A1 immunofluorescence. **(f)** Quantitative summary.

SA-β-galactosidase staining showed that H_2_O_2_ treatment (200 μM, 2 h) significantly increased the percentage of senescent cells from 8.3% ± 2.1% (Control) to 67.5% ± 8.4% (Model). Exosome treatment dose-dependently reduced the proportion of SA-β-gal-positive cells to 52.3% ± 6.7% (25 μg/mL), 38.6% ± 5.9% (50 μg/mL), and 21.4% ± 4.3% (100 μg/mL), respectively (Figure 2a). CCK-8 assay demonstrated that H_2_O_2_ treatment markedly inhibited hDPCs proliferation, reducing cell viability to 54.2% ± 6.8% at 48 h. Exosome treatment restored cell proliferative capacity in a dose-dependent manner, with the high-dose group (100 μg/mL) achieving 92.6% ± 5.4% viability at 72 h (Figure 2b).

Western blot analysis revealed that H_2_O_2_ treatment significantly decreased COL17A1 protein expression (0.32 ± 0.08-fold vs. Control) while increasing senescence markers p16^INK4a (3.85 ± 0.62-fold) and p21^Cip1 (2.94 ± 0.47-fold). Exosome treatment (100 μg/mL) reversed these changes, restoring COL17A1 to 0.89 ± 0.12-fold and reducing p16 and p21 to 1.45 ± 0.28-fold and 1.32 ± 0.24-fold, respectively (Figure 2c). qRT-PCR results confirmed that exosomes significantly upregulated COL17A1 mRNA (2.67 ± 0.38-fold vs. H_2_O_2_ group), along with hair follicle stem cell markers KRT15 (2.15 ± 0.31-fold vs. H_2_O_2_ group) and CD200 (1.89 ± 0.27-fold vs. H_2_O_2_ group) (Figure 2d). Immunofluorescence staining showed enhanced COL17A1 expression with prominent membrane localization in exosome-treated cells (Figure 2e). Quantitative analysis of all results is presented in Figure 2f. All fold-change values for the exosome-treated groups were calculated relative to the H_2_O_2_-treated group unless otherwise stated.

### 3.3 Exosomes promote Ex vivo human hair follicle growth

**FIGURE 3:**
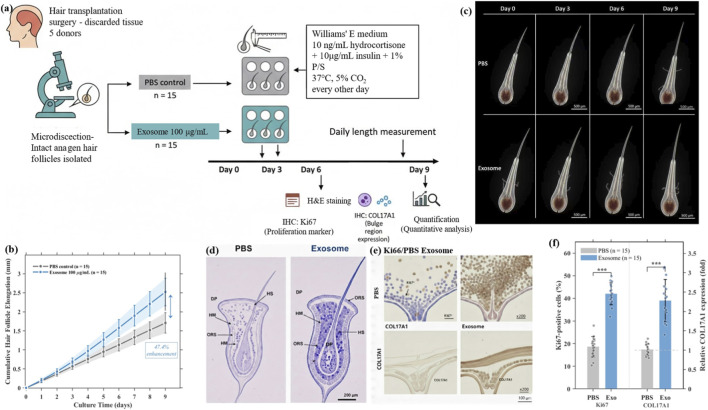
Exosomes promote *ex vivo* human hair follicle growth. **(a)** Experimental design. **(b)** Follicle elongation curve. **(c)** Representative images at days 0–9. **(d)** H&E staining. **(e)** Ki67 and COL17A1 IHC. **(f)** Quantification.

To validate the effects of exosomes under more physiologically relevant conditions, we established an *ex vivo* human hair follicle organ culture model. Intact anagen hair follicles were isolated and randomly assigned to PBS control or exosome treatment (100 μg/mL) groups (n = 15 per group from 5 donors). The experimental design is illustrated in Figure 3a.

Hair follicle length was measured daily for 9 days. The results showed that exosome-treated follicles exhibited significantly faster growth rates compared to the control group. The average daily elongation rate was 0.28 ± 0.04 mm/day in the exosome group versus 0.19 ± 0.03 mm/day in the PBS group (P < 0.001). By day 9, the cumulative length increase was 2.52 ± 0.36 mm in the exosome group compared to 1.71 ± 0.27 mm in the control group, representing a 47.4% enhancement (Figure 3b). Representative images clearly demonstrated the morphological differences between the two groups at days 0, 3, 6, and 9 (Figure 3c).

H&E staining revealed that exosome-treated follicles maintained intact structure with well-organized hair matrix cells, while some control follicles showed signs of premature catagen entry by day 9 (Figure 3d). Immunohistochemistry showed that Ki67-positive proliferating cells increased from 18.5% ± 3.6% (PBS) to 42.3% ± 5.8% (Exosome) in the hair matrix region. COL17A1 expression in the bulge region was enhanced 2.34 ± 0.42-fold in exosome-treated follicles (Figure 3e). Quantitative analysis is presented in Figure 3f.

### 3.4 Exosomes accelerate hair regeneration in C57BL/6 mice

**FIGURE 4:**
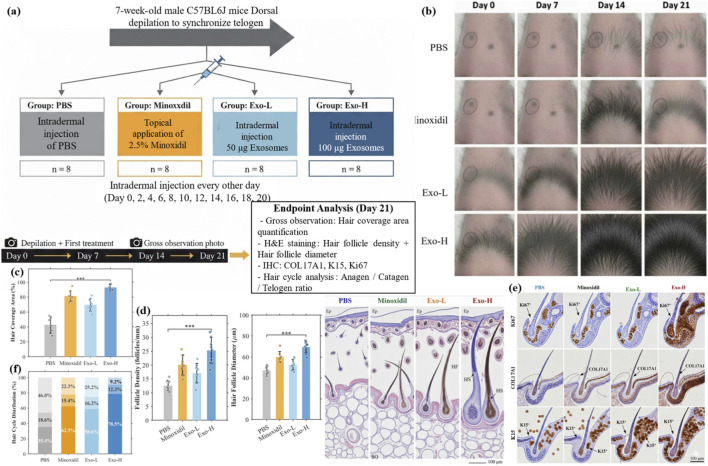
Exosomes accelerate hair regeneration in C57BL/6 mice. **(a)** Experimental timeline. **(b)** Dorsal skin photographs at days 0–21. **(c)** Hair coverage quantification. **(d)** H&E histology. **(e)** COL17A1, K15, and Ki67 IHC. **(f)** Hair cycle analysis.

To evaluate the hair growth-promoting effects of exosomes *in vivo*, we employed the well-established C57BL/6 mouse depilation-induced hair regeneration model. Mice were randomly divided into four groups (n = 8 per group): PBS, Minoxidil (2.5%), Exo-L (50 μg), and Exo-H (100 μg). The experimental timeline and treatment protocol are illustrated in Figure 4a.

Gross observation revealed striking differences in hair regeneration among groups. At day 7, all groups showed minimal hair growth with predominantly pink skin (telogen). By day 14, the Exo-H group exhibited significant darkening of the skin (indicating anagen entry) and visible hair growth, while the PBS group remained largely in telogen. At day 21, the Exo-H group achieved nearly complete hair coverage (92.4% ± 5.6%), compared to Minoxidil (78.3% ± 7.2%), Exo-L (71.5% ± 8.4%), and PBS (45.6% ± 9.8%) groups (Figures 4b,c).

Histological analysis by H&E staining revealed that exosome treatment significantly increased hair follicle density from 12.3 ± 2.1 follicles/mm (PBS) to 24.6 ± 3.8 follicles/mm (Exo-H). Hair follicle diameter also increased from 45.2 ± 6.7 μm (PBS) to 68.4 ± 8.2 μm (Exo-H) (Figure 4d). Immunohistochemical analysis demonstrated elevated expression of COL17A1 (2.78 ± 0.45-fold), K15 (2.23 ± 0.38-fold), and Ki67 (proliferation index: 52.4% ± 7.3% vs. 21.6% ± 4.5%) in the Exo-H group compared to PBS (Figure 4e). Notably, Ki67 positivity remained confined to the hair follicle epithelium and showed no evidence of aberrant proliferation in interfollicular epidermis or dermal compartments across all treatment groups, indicating that exosome-induced Wnt activation did not promote dysregulated tissue proliferation within the 21-day observation period. Hair cycle analysis showed that exosome treatment shifted the follicle population toward anagen: Exo-H group had 78.5% ± 6.8% anagen, 12.3% ± 3.2% catagen, and 9.2% ± 2.8% telogen follicles, whereas PBS group had 35.4% ± 7.5% anagen, 18.6% ± 4.1% catagen, and 46.0% ± 8.2% telogen follicles (Figure 4f).

### 3.5 Exosomes activate the Wnt/β-catenin signaling pathway

**FIGURE 5:**
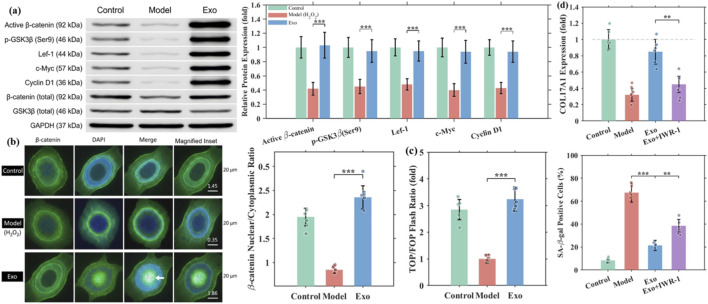
Exosomes activate the Wnt/β-catenin pathway. **(a)** Western blot of pathway components. **(b)** β-catenin immunofluorescence. **(c)** TOP/FOP Flash assay. **(d)** IWR-1 inhibition experiments.

The Wnt/β-catenin signaling pathway is essential for hair follicle morphogenesis, cycling, and regeneration (Liu J. et al., 2022; Choi, 2020). We investigated whether exosomes activate this pathway to promote hair growth. Western blot analysis showed that exosome treatment (100 μg/mL, 48 h) significantly increased active (non-phosphorylated) β-catenin levels (2.45 ± 0.36-fold vs. Model), elevated phospho-GSK3β at Ser9 (2.12 ± 0.28-fold, indicating GSK3β inactivation), and upregulated downstream targets including Lef-1 (1.98 ± 0.25-fold), c-Myc (2.34 ± 0.32-fold), and Cyclin D1 (2.18 ± 0.29-fold) (Figure 5a).

Immunofluorescence staining demonstrated that in the model group, β-catenin was predominantly localized in the cytoplasm and cell membrane. Exosome treatment induced marked nuclear translocation of β-catenin, with nuclear/cytoplasmic ratio increasing from 0.35 ± 0.08 to 1.86 ± 0.24 (Figure 5b). TOP/FOP Flash reporter assay confirmed that exosomes enhanced Wnt pathway transcriptional activity by 3.24 ± 0.45-fold compared to the model group (Figure 5c).

To verify the functional requirement of Wnt pathway activation, we performed pathway inhibition experiments. Pretreatment with IWR-1 (10 μM), a tankyrase inhibitor that stabilizes Axin and promotes β-catenin degradation, significantly attenuated the exosome-induced effects. IWR-1 reduced COL17A1 mRNA upregulation from 2.67 ± 0.38-fold to 1.42 ± 0.21-fold and partially restored the senescent phenotype (SA-β-gal positive cells: 38.5% ± 5.6% vs. 21.4% ± 4.3% without IWR-1) (Figure 5d). These results indicate that the Wnt/β-catenin pathway is a key mediator of exosome biological functions.

### 3.6 Exosomal miR-21-5p promotes COL17A1 expression by targeting DKK2

**FIGURE 6:**
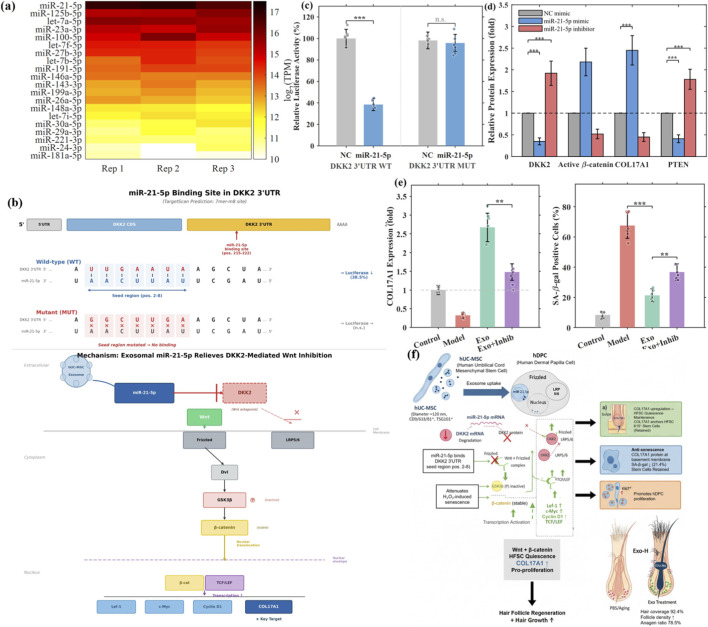
Exosomal miR-21-5p promotes COL17A1 expression by targeting DKK2. **(a)** Heatmap of the top 20 abundant miRNAs in hUC-MSC exosomes. **(b)** Predicted binding site of miR-21-5p in the DKK2 3′UTR and pathway schematic. **(c)** Dual-luciferase reporter assay validating miR-21-5p targeting of DKK2 (WT vs. MUT). **(d)** Western blot of DKK2, active β-catenin, COL17A1, and PTEN after miR-21-5p mimic or inhibitor transfection. **(e)** Rescue experiments: effects of miR-21-5p inhibitor on exosome-mediated COL17A1 upregulation and SA-β-gal staining. **(f)** Proposed mechanistic model of the miR-21-5p/DKK2/Wnt/β-catenin/COL17A1 axis. Data are mean ± SD (n = 3).

To elucidate the molecular mechanism underlying exosome-mediated Wnt pathway activation and COL17A1 upregulation, we performed small RNA sequencing on hUC-MSC-derived exosomes. Heatmap analysis revealed the expression profile of the top 20 most abundant miRNAs (Figure 6a). Among them, miR-21-5p was the most highly expressed, followed by miR-125b-5p, let-7a-5p, and others (Table 2). The complete miRNA expression data are provided in Supplementary Table S3.

**TABLE 2:** Top 10 abundant miRNAs in hUC-MSC exosomes and predicted targets.

Rank	miRNA	Abundance (TPM)	Predicted target genes	Functional relevance
1	miR-21-5p	15,842 ± 2,136	**DKK2**, PTEN, PDCD4, SPRY1	**Wnt activation (targeting DKK2)**
2	miR-125b-5p	8,756 ± 1,245	TP53, BAK1, BMF	Anti-apoptosis
3	let-7a-5p	6,423 ± 987	HMGA2, LIN28, KRAS	Cell proliferation
4	miR-100-5p	4,215 ± 634	mTOR, IGF1R, FGFR3	Metabolic regulation
5	miR-29a-3p	3,876 ± 521	COL1A1, COL3A1, MCL1	ECM remodeling
6	miR-23a-3p	3,124 ± 478	APAF1, PTEN, SIRT1	Anti-apoptosis
7	miR-221-3p	2,856 ± 412	CDKN1B, KIT, PTEN	Cell cycle regulation
8	miR-24-3p	2,534 ± 389	BIM, FAF1, CDKN1B	Anti-apoptosis
9	miR-199a-3p	2,145 ± 324	mTOR, MET, CAV1	Proliferation/migration
10	miR-126-3p	1,987 ± 298	SPRED1, PIK3R2, VEGFA	Angiogenesis

TPM, transcripts per million. Bold indicates the key miRNA, and target validated in this study.

Using TargetScan and miRDB databases, we performed target prediction analysis focusing on Wnt pathway regulators. Bioinformatics analysis identified DKK2 (Dickkopf-2), a secreted Wnt antagonist belonging to the same family as DKK1, as a validated target of miR-21-5p. Previous studies have suggested that miR-21 targets DKK2 to activate Wnt/β-catenin signaling in cancer cells, based on bioinformatic prediction and protein expression analysis (Kawakita et al., 2014). DKK2, like DKK1, antagonizes canonical Wnt signaling by binding to LRP5/6 co-receptors (Song et al., 2018; Figure 6b). GO enrichment analysis revealed that predicted targets were significantly enriched in “negative regulation of Wnt signaling pathway” (GO:0030178, P = 2.3 × 10^−8^) and “cell aging” (GO:0007569, P = 4.6 × 10^−6^). KEGG pathway analysis highlighted enrichment in “Wnt signaling pathway” (hsa04310, P = 1.8 × 10^−7^) (Supplementary Tables S4-S5).

Dual-luciferase reporter assay was performed to validate the direct interaction between miR-21-5p and DKK2 3′UTR. Co-transfection of miR-21-5p mimic with wild-type DKK2 3′UTR reporter in HEK293T cells significantly reduced luciferase activity to 38.5% ± 5.7% compared to negative control mimic. This suppression was abolished when the predicted binding site was mutated (Figure 6c).

Functional validation in hDPCs showed that transfection of miR-21-5p mimic (50 nM) recapitulated the effects of exosomes: DKK2 protein was reduced to 0.35 ± 0.08-fold, active β-catenin increased 2.18 ± 0.32-fold, and COL17A1 was upregulated 2.45 ± 0.34-fold compared to negative control. Conversely, miR-21-5p inhibitor (100 nM) elevated DKK2 (1.92 ± 0.28-fold), decreased active β-catenin (0.52 ± 0.11-fold), and reduced COL17A1 (0.45 ± 0.10-fold) (Figure 6d). To evaluate whether PTEN, another validated miR-21-5p target, contributed to the observed phenotype, we assessed PTEN protein levels in the same transfection conditions. miR-21-5p mimic reduced PTEN protein to 0.41 ± 0.09-fold of NC mimic, while miR-21-5p inhibitor increased PTEN to 1.78 ± 0.23-fold of NC inhibitor (Figure 6d), confirming that PTEN is regulated by miR-21-5p in hDPCs. However, as PTEN primarily suppresses the PI3K-Akt pathway rather than directly antagonizing Wnt/β-catenin signaling, its modulation is unlikely to account for the DKK2-dependent Wnt activation and COL17A1 upregulation documented in our system. Parallel contributions of PTEN to the broader anti-senescence phenotype through PI3K-Akt-mediated mechanisms cannot be excluded and are acknowledged as a limitation warranting further investigation.

Importantly, rescue experiments demonstrated that miR-21-5p inhibitor partially blocked exosome-mediated effects. In cells transfected with miR-21-5p inhibitor followed by exosome treatment, COL17A1 mRNA upregulation was attenuated from 2.67 ± 0.38-fold (Exo alone) to 1.48 ± 0.22-fold (Exo + inhibitor), and the anti-senescence effect was reduced (SA-β-gal positive cells: 36.8% ± 5.4% vs. 21.4% ± 4.3%) (Figure 6e). These results confirm that miR-21-5p is a key functional component of exosomes mediating Wnt pathway activation. It should be noted, however, that the partial nature of the rescue (COL17A1 upregulation attenuated from 2.67-fold to 1.48-fold but not abolished) is consistent with parallel contributions from additional miR-21-5p targets such as PTEN or PDCD4, none of which were directly investigated in the present study. This multi-target complexity is acknowledged as a limitation and warrants further investigation. The partial but significant attenuation by the miR-21-5p inhibitor further indicates that the biological activity of the exosome preparation is at least partly dependent on intracellular miRNA function, rather than being attributable solely to non-miRNA components such as exosomal surface proteins or lipid membrane effects.

Based on these findings, we propose a working model in which hUC-MSC-derived exosomes deliver miR-21-5p into hDPCs, where miR-21-5p suppresses DKK2 expression, thereby relieving Wnt pathway inhibition. This is proposed to lead to GSK3β inactivation, β-catenin stabilization and nuclear translocation, and subsequent transcriptional upregulation of target genes including COL17A1. Enhanced COL17A1 expression is associated with maintenance of hair follicle regenerative capacity and attenuation of cellular senescence (Figure 6f). We emphasize that while the key nodes of this axis are supported by direct experimental evidence—including dual-luciferase validation of miR-21-5p/DKK2 interaction, DKK2 siRNA recapitulation of exosome effects, TOPflash reporter confirmation of Wnt activation, and IWR-1 attenuation of COL17A1 upregulation—the transcriptional link between β-catenin/TCF-LEF and the COL17A1 promoter in hDPCs remains to be directly demonstrated.

### 3.7 DKK2 directly regulates COL17A1 expression in a context-dependent manner in hDPCs

**FIGURE 7:**
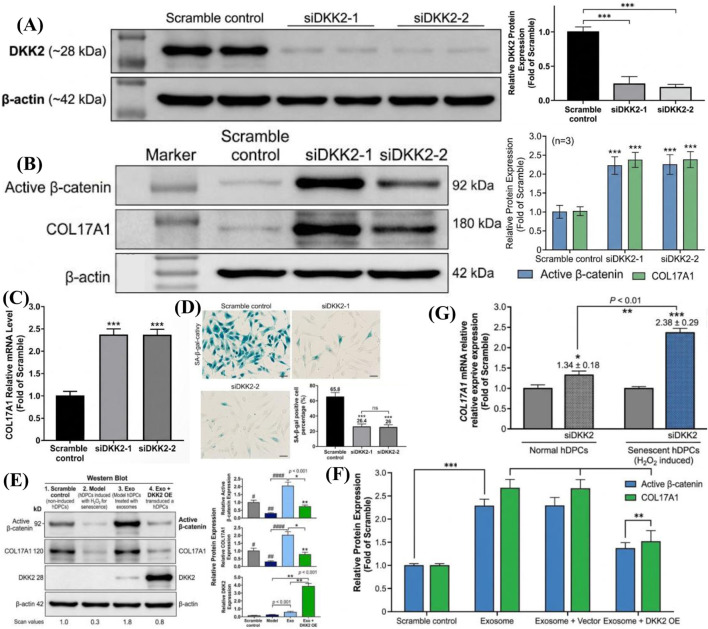
DKK2 directly regulates COL17A1 expression in senescent hDPCs. **(A)** Western blot confirming DKK2 knockdown efficiency of siDKK2-1 and siDKK2-2 in senescent hDPCs. **(B)** Western blot of active β-catenin and COL17A1 following DKK2 siRNA knockdown in senescent hDPCs. **(C)** qRT-PCR of COL17A1 mRNA following DKK2 knockdown. **(D)** SA-β-gal staining following DKK2 knockdown in senescent hDPCs. **(E,F)** Western blot of active β-catenin and COL17A1 in exosome-treated senescent hDPCs with or without DKK2 overexpression. **(G)** Comparison of COL17A1 mRNA response to DKK2 knockdown in normal versus senescent hDPCs. Data are mean ± SD (n = 3). **P < 0.01, ***P < 0.001.

To directly establish the functional role of DKK2 in the senescent hDPC context and resolve its context-dependent activity, we performed DKK2 siRNA knockdown and overexpression experiments in both normal and H_2_O_2_-induced senescent hDPCs. These experiments were performed using the same n = 3 independent replicates and donor pooling design as described for the original *in vitro* experiments (hDPCs pooled from 2 donors per replicate, 6 donors total per condition), with all replicates derived from donors independent of those used in the initial senescence experiments. Two independent siRNAs (siDKK2-1 and siDKK2-2) achieved protein knockdown efficiencies of 78.3% ± 5.4% and 82.1% ± 6.7%, respectively, compared to scramble control (Figure 7A).

In senescent hDPCs, DKK2 silencing recapitulated the key effects of exosome treatment: active β-catenin was increased 2.24 ± 0.31-fold, COL17A1 mRNA was upregulated 2.38 ± 0.29-fold, and SA-β-gal-positive cells were reduced from 65.8% ± 6.3% to 26.4% ± 4.8% compared to scramble control (Figures 7B–D). These effects were consistent across both siRNA sequences, excluding off-target contributions. Conversely, DKK2 overexpression in exosome-treated senescent hDPCs partially reversed the exosome-induced COL17A1 upregulation (from 2.67 ± 0.38-fold to 1.52 ± 0.24-fold) and attenuated active β-catenin accumulation (from 2.31 ± 0.28-fold to 1.38 ± 0.19-fold), confirming that DKK2 suppression is functionally required for exosome-mediated COL17A1 regulation (Figures 7E,F).

Notably, in normal (non-senescent) hDPCs, DKK2 knockdown produced a comparatively modest effect on COL17A1 expression (1.34 ± 0.18-fold increase vs. scramble control), markedly less pronounced than that observed in senescent cells (2.38 ± 0.29-fold, P < 0.01, Figure 7G). This differential response confirms that the inhibitory role of DKK2 on Wnt/β-catenin signaling predominates specifically under oxidative stress conditions, consistent with the altered co-receptor landscape previously proposed to explain DKK2 dual functionality (Mao et al., 2002; Mao and Niehrs, 2003). Collectively, these data provide direct experimental evidence that DKK2 functions as a context-dependent Wnt antagonist in senescent hDPCs, and that its suppression by miR-21-5p is a functionally required step in the exosome-mediated COL17A1 regulatory axis.

## 4 Discussion

Hair follicle aging, characterized by follicle miniaturization, hair thinning, and eventual hair loss, represents a significant clinical challenge with limited therapeutic options. In this study, we provide evidence that hUC-MSC-derived exosomes attenuate hair follicle-associated cellular senescence and promote hair regeneration through a mechanism involving COL17A1 upregulation. Mechanistically, our data support a working model in which exosomal miR-21-5p targets the Wnt antagonist DKK2, thereby activating the Wnt/β-catenin signaling pathway and subsequently enhancing COL17A1 expression, though direct transcriptional evidence at the promoter level remains to be established. These findings provide novel insights into the molecular mechanisms of exosome-mediated hair follicle rejuvenation and suggest a promising therapeutic strategy for scalp anti-aging treatment.

### 4.1 Exosomes as a novel approach for hair follicle rejuvenation

Our study provides comprehensive evidence supporting the anti-senescence and hair growth-promoting effects of MSC-derived exosomes across three experimental levels: *in vitro* hDPCs culture, *ex vivo* human hair follicle organ culture, and *in vivo* mouse hair regeneration model. It should be noted that the H_2_O_2_-induced senescence model employed here recapitulates key hallmarks of cellular senescence, including SA-β-gal positivity, p16^INK4a^ and p21^Cip1^ upregulation, and COL17A1 downregulation, that are also observed in naturally aged hair follicle cells. However, H_2_O_2_ treatment models acute oxidative injury rather than replicative or chronological aging, and the transcriptomic and epigenetic landscapes of these two senescence modalities may differ. The equivalence of COL17A1 downregulation in this model to its loss in aged HFSCs *in vivo* therefore represents an assumption rather than a demonstrated fact, and comparative studies using replicative senescence models or primary cells from aged donors will be necessary to fully validate the translational relevance of our findings. In *ex vivo* cultured human hair follicles, exosomes enhanced the elongation rate by 47.4% over 9 days. In the C57BL/6 mouse model, high-dose exosome treatment achieved 92.4% hair coverage compared to 45.6% in the PBS control group at day 21, outperforming even the minoxidil positive control (78.3%). It should be acknowledged, however, that the C57BL/6 depilation model primarily interrogates the telogen-to-anagen transition rather than age-related HFSC exhaustion. The enhanced hair regeneration observed in this model therefore reflects restoration of hair cycle competence rather than direct reversal of chronological aging *per se*. The biological rationale for this translational inference is that senescent HFSCs exhibit impaired anagen entry capacity, and that restoration of COL17A1 expression and Wnt pathway activity—as demonstrated in our *in vitro* and *ex vivo* systems—is mechanistically associated with recovery of this capacity. Nevertheless, the use of a hair cycling model to make inferences about an aging mechanism represents a translational assumption that future studies employing aged mouse models or humanized skin xenograft systems will need to formally address.

These results are consistent with and extend previous studies on exosomes in hair regeneration. Chen et al. (2020) reported that human dermal papilla cell-derived exosomes promote hair growth through miR-140-5p-mediated BMP signaling modulation in an anagen-inducing mouse model. Kim et al. (2022) demonstrated that bovine colostrum-derived exosomes accelerate hair regeneration via Wnt/β-catenin pathway activation. However, these studies did not address the aging-related decline of hair follicle function (Hu et al., 2020; Wu et al., 2021; Tang et al., 2023; Lee E. et al., 2024). Our study is among the first to establish a mechanistic link between MSC-exosome miRNA cargo and COL17A1 regulation in the context of hair follicle aging, and demonstrate that exosomes can reverse the age-associated downregulation of this critical protein.

### 4.2 COL17A1 as a central target for hair follicle anti-aging

COL17A1 (type XVII collagen, also known as BP180) is a transmembrane protein that anchors hair follicle stem cells (HFSCs) to the basement membrane at the bulge region. The seminal work by Matsumura et al. (2016) published in Science revealed that COL17A1 proteolysis triggers HFSC aging and hair follicle miniaturization. They demonstrated that aged HFSCs lose COL17A1 expression, detach from the basement membrane, differentiate into epidermal keratinocytes, and are eventually eliminated through transepidermal elimination (Zhang et al., 2021; Shin et al., 2020). This finding established COL17A1 as a master regulator of HFSC homeostasis and a central target for anti-aging interventions (Matsumura et al., 2021; Han et al., 2022).

Our study provides compelling evidence that COL17A1 can be exogenously upregulated by MSC-derived exosomes. We observed that exosome treatment increased COL17A1 protein levels by approximately 2.78-fold (from 0.32 ± 0.08 to 0.89 ± 0.12 relative to control) in senescent hDPCs and enhanced COL17A1 expression in the bulge region of both *ex vivo* human follicles and *in vivo* mouse skin. Importantly, this upregulation was accompanied by increased expression of HFSC markers K15 and CD200, suggesting that exosomes may help maintain the hair follicle regenerative microenvironment, as indirectly evidenced by the upregulation of bulge-associated markers K15 and CD200 in both *ex vivo* human follicles and *in vivo* mouse skin (Liu F. et al., 2024). These findings suggest that exosome-based therapy could potentially counteract the COL17A1 decline observed during natural aging, offering a new avenue for hair follicle rejuvenation.

### 4.3 The miR-21-5p/DKK2/Wnt/COL17A1 regulatory axis

A key contribution of our study is the elucidation of the molecular mechanism by which exosomes upregulate COL17A1. Through small RNA sequencing, we identified miR-21-5p as the most abundant miRNA in hUC-MSC exosomes (15,842 TPM), consistent with previous reports showing miR-21-5p enrichment in MSC-derived extracellular vesicles across multiple tissue sources including bone marrow, adipose tissue, and umbilical cord (Yang et al., 2024; Xie et al., 2022).

miR-21-5p is a well-characterized oncomiR with established roles in cell survival, proliferation, and anti-apoptosis, with numerous validated targets including PTEN, PDCD4, and SPRY1. Among these, DKK2 was prioritized for functional validation based on two converging lines of evidence: first, DKK2 is the most direct predicted miR-21-5p target with a well-established role as a canonical Wnt/β-catenin pathway antagonist; while PTEN can modulate β-catenin stability through GSK-3β-independent mechanisms and SPRY1 intersects with receptor tyrosine kinase signaling that cross-talks with the Wnt pathway, neither functions as a direct extracellular Wnt antagonist in the manner of DKK2, making it the most biologically plausible mediator of the Wnt activation phenotype observed in our system; second, Kawakita et al. (2014) previously reported miR-21-mediated DKK2 suppression in the context of Wnt pathway activation, providing prior experimental support for this interaction. Our dual-luciferase reporter assay provides direct experimental validation of the miR-21-5p/DKK2 interaction in the context of hair follicle biology. DKK2 (Dickkopf-2) is a secreted glycoprotein that, like its family member DKK1, can antagonize canonical Wnt signaling by interfering with the Wnt-Frizzled-LRP signaling complex (Song et al., 2018; Lee S. B. et al., 2024). Our dual-luciferase reporter assay confirmed direct binding of miR-21-5p to the DKK2 3′UTR, and functional experiments demonstrated that miR-21-5p mimic reduced DKK2 expression while increasing active β-catenin and COL17A1 levels.

It is important to note that DKK2 exhibits context-dependent dual functionality in Wnt signaling regulation, which distinguishes it from DKK1, a canonical Wnt antagonist. While Song et al. (2018) demonstrated that DKK2 suppresses Wnt/β-catenin signaling in plantar skin dermis and epidermis, thereby inhibiting hair follicle formation, Lee S. B. et al. (2024) reported that exogenous DKK2 treatment promotes Wnt/β-catenin signaling and hair growth in normal human dermal papilla cells. This apparent paradox likely reflects the cell-type and microenvironment-dependent nature of DKK2 function, which has been attributed to differences in co-receptor availability, particularly the relative expression levels of Kremen proteins and LRP5/6 (Mao et al., 2002; Mao and Niehrs, 2003). In our experimental system, hDPCs were subjected to H_2_O_2_-induced oxidative stress, which may alter the receptor landscape and shift DKK2 toward an inhibitory role. Moreover, our functional data consistently support a Wnt-antagonistic role for DKK2 under our experimental conditions: miR-21-5p-mediated DKK2 suppression led to increased active β-catenin levels, enhanced TOPflash reporter activity, and upregulated Wnt target genes, while these effects were attenuated by the Wnt pathway inhibitor IWR-1. To directly address this question, we performed DKK2 siRNA knockdown and overexpression experiments in both normal and H_2_O_2_-induced senescent hDPCs (Figure 7). DKK2 silencing in senescent hDPCs recapitulated the pro-regenerative effects of exosome treatment, while DKK2 overexpression partially reversed exosome-induced COL17A1 upregulation and β-catenin activation, collectively confirming that DKK2 functions as a Wnt antagonist under oxidative stress conditions in our experimental system. The comparatively modest effect of DKK2 knockdown in normal hDPCs further supports the context-dependent nature of DKK2 function, consistent with the altered co-receptor landscape previously proposed to explain DKK2 dual functionality (Mao et al., 2002; Mao and Niehrs, 2003). These findings provide direct experimental evidence resolving the apparent paradox between our results and those of Lee S. B. et al. (2024), and confirm that DKK2 suppression is a functionally required step in exosome-mediated COL17A1 regulation specifically within the senescent microenvironment.

The link between Wnt/β-catenin signaling and COL17A1 expression has been suggested by previous studies (Zhao et al., 2022; Liang et al., 2023). Watanabe et al. (2017) reported that Wnt signaling positively regulates COL17A1 transcription in interfollicular epidermis. We acknowledge that this transcriptional relationship was originally described in interfollicular epidermis rather than in dermal papilla cells, and that direct evidence for β-catenin/TCF-LEF binding to the COL17A1 promoter in hDPCs—such as chromatin immunoprecipitation or promoter reporter assays—was not obtained in the present study. Nevertheless, our functional data provide convergent indirect evidence supporting this regulatory relationship in hDPCs: exosome treatment increased active β-catenin and nuclear translocation (Figures 5a,b), enhanced TOPflash reporter activity (Figure 5c), upregulated canonical Wnt target genes (c-Myc, Cyclin D1, LEF-1), and concurrently increased COL17A1 mRNA and protein levels; these effects were attenuated by the Wnt inhibitor IWR-1 (Figure 5d) and recapitulated by DKK2 siRNA knockdown (Figure 7). The absence of direct promoter-level evidence is acknowledged as a limitation, and future studies employing ChIP-qPCR or COL17A1 promoter-luciferase reporter assays in hDPCs will be necessary to formally establish the transcriptional mechanism. The use of IWR-1, a tankyrase inhibitor that promotes β-catenin degradation, partially blocked the exosome effects, confirming the functional requirement of Wnt pathway activation (Han et al., 2022).

Based on our findings, we propose the following mechanistic model: hUC-MSC-derived exosomes deliver miR-21-5p into recipient hDPCs, where miR-21-5p suppresses DKK2 expression at the post-transcriptional level. Reduced DKK2 relieves the inhibition on Wnt/LRP6 signaling, leading to GSK3β inactivation, β-catenin stabilization, and nuclear translocation. Nuclear β-catenin forms complexes with TCF/LEF transcription factors to activate target genes, including COL17A1. Enhanced COL17A1 expression maintains HFSC anchoring to the basement membrane and preserves stem cell identity, ultimately attenuating cellular senescence and promoting hair regeneration.

### 4.4 Clinical translation potential

Our findings have significant implications for clinical translation in the field of aesthetic medicine and dermatology. Current FDA-approved treatments for hair loss, including minoxidil and finasteride, have limitations in efficacy, require continuous use, and are associated with side effects such as scalp irritation (minoxidil) and sexual dysfunction (finasteride) (Kaiser et al., 2023; Gupta et al., 2022). Exosome-based therapy offers several potential advantages.

First, MSC-derived exosomes have favorable safety profiles. Unlike cell-based therapies, exosomes are acellular, non-replicating, and have low immunogenicity due to their small size and lack of MHC molecules. Multiple clinical trials have demonstrated the safety of MSC-exosome administration in various conditions (Bai et al., 2024; Lee E. et al., 2024). We acknowledge, however, that miR-21-5p is a well-characterized oncomiR, and the potential oncogenic implications of its repeated delivery warrant careful consideration. Several factors mitigate this concern in our therapeutic context: intradermal injection confines delivery to the local scalp microenvironment with minimal systemic exposure; exosome-delivered miR-21-5p represents a transient, non-integrating cargo that is fundamentally distinct from the sustained endogenous miR-21 overexpression that drives tumor progression; and Ki67 staining in our 21-day mouse study showed no evidence of aberrant proliferation. Beyond miR-21-5p, the oncogenic potential of Wnt/β-catenin pathway activation also warrants consideration. It is important to note, however, that Wnt signaling undergoes physiological cyclical activation and suppression during normal hair follicle cycling, and that the exosome-induced activation observed here is analogous to the endogenous anagen-phase Wnt activation rather than constitutive pathway dysregulation. The reversibility of this activation was further demonstrated by the IWR-1 inhibition experiments (Figure 5d), which showed that pharmacological Wnt blockade readily attenuated the exosome-induced effects. Ki67 quantification in our 21-day mouse study confirmed that proliferative activity remained confined to the hair follicle epithelium with no evidence of aberrant proliferation in surrounding tissues (Figure 4e). Nevertheless, long-term safety monitoring beyond the 21-day observation window, including systematic tumor surveillance and assessment of sustained Wnt pathway activity, will be an essential prerequisite for clinical translation. Second, exosomes can be standardized, stored, and administered as an “off-the-shelf” product, overcoming the logistical challenges of autologous cell therapies (Kost et al., 2022). Third, our data suggest that exosomes may outperform minoxidil in promoting hair regeneration under the experimental conditions employed, as the high-dose exosome group achieved superior hair coverage compared to the minoxidil control. We note, however, that these results were obtained under optimized laboratory conditions and that the generalizability of this comparison to clinical settings will require validation in larger, more heterogeneous study populations.

The intradermal injection route used in our mouse study shares procedural similarities with established clinical scalp delivery approaches such as mesotherapy and platelet-rich plasma injection protocols (Yuan et al., 2024), suggesting potential translational compatibility. However, direct translation from murine to human dosing cannot be assumed without allometric scaling, pharmacokinetic modeling, and tissue distribution data in larger animal models. The dosing regimen employed here (100 μg exosomes every other day in mice) therefore serves as a preliminary starting point for preclinical dose-finding studies rather than a clinical dose reference, and substantial further optimization will be required before human application can be considered.

### 4.5 Limitations and future directions

Several limitations of this study should be acknowledged. First, regarding study population representativeness, the sample sizes employed in this study (n = 3 independent replicates per group for *in vitro* experiments; n = 8 per group for the *in vivo* mouse study) are relatively small, and the *in vitro* experiments were performed with a donor pooling strategy that may underestimate inter-individual biological variability. Rather than reporting *post hoc* power estimates calculated from observed effect sizes, which are methodologically circular, we present complete effect sizes and standard deviations throughout to allow readers to assess the strength and precision of the evidence directly. Independent replication in larger cohorts will be necessary to confirm the robustness of these findings across broader biological contexts. All donors were of Chinese/East Asian ethnicity, which represents a substantive biological limitation given known differences in hair follicle morphology, density, and potentially Wnt pathway activity across ethnic groups. Furthermore, the *in vitro* experiments used hDPCs rather than directly isolated bulge HFSCs; while hDPCs serve as the primary mediators of dermal-epithelial crosstalk governing HFSC activation, extrapolation to bulge HFSC biology should be made with caution. The upregulation of K15 and CD200 in *ex vivo* and *in vivo* tissues (Figure 4e) provides indirect evidence of effects extending to the HFSC niche, but direct analysis of LGR5^+^ or SOX9^+^ populations remains necessary.

Second, regarding *in vivo* model limitations, only male C57BL/6J mice were employed, which represents a substantive limitation given that androgenetic alopecia involves well-established sex-specific differences in androgen signaling and treatment response. It should be noted, however, that the depilation-induced hair cycle synchronization protocol used here bypasses androgen-mediated follicular miniaturization, and the molecular readouts assessed (COL17A1, K15, Ki67) reflect exosome effects on the hair cycle machinery rather than on androgen-sensitive pathways; sex-balanced cohorts and androgen-exposed models will therefore be a priority in future translational studies. Additionally, the C57BL/6 depilation model primarily interrogates the telogen-to-anagen transition rather than age-related HFSC exhaustion, and does not fully recapitulate progressive follicular miniaturization characteristic of chronological aging. Nevertheless, the core mechanistic conclusions of this study are established through the *in vitro* and *ex vivo* experiments; the *in vivo* component serves to confirm hair regeneration at the whole-organ level, a readout for which the depilation model is well-validated and widely accepted. The functional specificity of exosomal miRNA cargo is supported by three converging lines of evidence: RNase A protection confirmed intraluminal miR-21-5p encapsulation (Supplementary Figure S1); miR-21-5p inhibitor rescue partially but significantly attenuated exosome-mediated COL17A1 upregulation and anti-senescence effects; and DKK2 siRNA knockdown independently recapitulated the exosome phenotype with comparable effect magnitudes, arguing against non-specific lipid bilayer contributions. Nevertheless, heat-inactivated exosome and exosome-depleted conditioned medium controls were not included, and contributions from soluble factors cannot be fully excluded; these controls should be incorporated in future studies.

Third, regarding mechanistic evidence strength, only one miR-21-5p target (DKK2) was validated, and other targets including PTEN, PDCD4, and SPRY1 may contribute through parallel mechanisms, consistent with the partial nature of the inhibitor rescue (2.67-fold attenuated to 1.48-fold). The transcriptional link between Wnt/β-catenin activation and COL17A1 upregulation was inferred from functional data rather than directly demonstrated at the chromatin level; ChIP or COL17A1 promoter reporter assays in hDPCs will be necessary to formally establish this relationship. Additionally, the H_2_O_2_-induced senescence model recapitulates key hallmarks of cellular senescence but models acute oxidative injury rather than replicative or chronological aging, and comparative studies with aged donor cells are warranted to validate translational relevance.

Fourth, long-term safety and efficacy of repeated exosome administration remain to be evaluated. The potential consequences of sustained Wnt/β-catenin activation and repeated miR-21-5p delivery beyond the 21-day observation window have not been assessed, and systematic histopathological examination and tumor surveillance will be essential prerequisites for clinical translation.

Finally, we acknowledge that the consistently large effect sizes across endpoints may in part reflect optimized experimental conditions. Not all endpoints showed complete normalization—the miR-21-5p inhibitor rescue was partial, IWR-1 restored SA-β-gal positivity to 38.5% rather than control levels, and Exo-L showed consistently intermediate effects compared to Exo-H—indicating dose-dependent rather than ceiling-level responses. All experimental data were reported in full and no endpoints were selectively excluded.

Future studies should address these limitations through development of humanized or aged mouse models, direct isolation and analysis of LGR5^+^/SOX9^+^ HFSCs, inclusion of sex-balanced cohorts and androgen-exposed models, ChIP-based promoter validation, and ultimately well-designed clinical trials to establish the safety and efficacy of exosome therapy for human hair loss.

## 5 Conclusion

In conclusion, this study provides evidence that human umbilical cord mesenchymal stem cell-derived exosomes effectively attenuate hair follicle cell senescence and promote hair regeneration through upregulating COL17A1 expression. Using a combination of *in vitro*, *ex vivo*, and *in vivo* approaches, we showed that exosome treatment reduced senescence-associated β-galactosidase-positive cells from 67.5% to 21.4% in H_2_O_2_-induced senescent hDPCs, enhanced human hair follicle elongation by 47.4% in organ culture, and achieved 92.4% hair coverage in C57BL/6 mice compared to 45.6% in controls. Mechanistically, we identified miR-21-5p as the most abundant miRNA in hUC-MSC exosomes and showed that it targets the Wnt antagonist DKK2, thereby relieving Wnt pathway inhibition and leading to β-catenin stabilization, nuclear translocation, and subsequent upregulation of COL17A1 expression. This proposed miR-21-5p/DKK2/Wnt/β-catenin/COL17A1 regulatory axis provides new insights into the molecular mechanisms underlying exosome-mediated tissue rejuvenation. Our findings suggest COL17A1 as a potential therapeutic target for hair follicle anti-aging and suggest that MSC-derived exosomes represent a promising cell-free strategy for scalp rejuvenation and hair loss treatment.

## Data Availability

The original contributions presented in the study are included in the article/Supplementary Material, further inquiries can be directed to the corresponding author.
