# Supplementary material for: Mesenchymal stem cell-derived exosomes promote scalp rejuvenation through type XVII collagen regulation via the miR-21-5p/DKK2/Wnt pathway
Source: Front Cell Dev Biol. 2026 Apr 22;14:1807877. doi: 10.3389/fcell.2026.1807877 (PMC13143841; doi:10.3389/fcell.2026.1807877)
Supplement: Supplementary file 1 [file DataSheet1.docx]

**Supplementary Materials**

**Table S1.** Antibody Information

| **Antibody** | **Host Species** | **Clone** | **Dilution (WB)** | **Dilution (IF/IHC)** | **Catalog No.** | **Manufacturer** | **Validated Applications** |
| --- | --- | --- | --- | --- | --- | --- | --- |
| CD9 | Rabbit | EPR23105-121 | 1:1000 | IF 1:500, IHC 1:100 | ab236630 | Abcam | WB, IF, IHC, Flow, IP |
| CD63 | Rabbit | EPR5702 | 1:1000 | IHC 1:250-1:2000 | ab134045 | Abcam | WB, IHC (not IF) |
| CD81 | Rabbit | EPR4244 | 1:700-1:1000 | N/A | ab109201 | Abcam | WB only |
| TSG101 | Rabbit | EPR7130(B) | 1:1000 | IF 1:50, IHC 1:300 | ab125011 | Abcam | WB, IF, IHC, Flow |
| Calnexin | Rabbit | Polyclonal | 1:250 | IF 1 µg/mL | ab22595 | Abcam | WB, IF, IHC, IP |
| COL17A1 | Rabbit | EPR18614 | 1:5000 | IF 1:100, IHC 1:100 | ab184996 | Abcam | WB, IF, IHC, IP |
| p16^INK4a | Rabbit | EPR1473 | 1:2000-1:4000 | IHC 1:100-1:250 | ab108349 | Abcam | WB, IHC, Flow (not IF) |
| p21^Cip1 | Rabbit | EPR362 | 1:1000 | IF 1:1000, IHC 1:100 | ab109520 | Abcam | WB, IF, IHC, Flow, IP |
| Keratin 15 (K15) | Rabbit | EPR1614Y | 1:10000 | IF 1:50, IHC validated | ab52816 | Abcam | WB, IF, IHC, Flow |
| CD200 | Rabbit | Polyclonal | N/A | IHC 1:200 | ab203887 | Abcam | IHC only |
| β-catenin | Rabbit | D10A8 | 1:1000 | IF 1:100-1:400, IHC 1:50-1:200 | #8480 | Cell Signaling Technology | WB, IF, IHC, Flow, ChIP |
| Active β-catenin (non-phospho Ser45) | Rabbit | D2U8Y | 1:1000 | IF 1:1600-1:6400, IHC 1:500-1:2000 | #19807 | Cell Signaling Technology | WB, IF, IHC, Flow |
| Phospho-GSK3β (Ser9) | Rabbit | Polyclonal | 1:1000 | N/A | #9336 | Cell Signaling Technology | WB only |
| GSK3β | Rabbit | 27C10 | 1:1000 | IHC 1:100 | #9315 | Cell Signaling Technology | WB, IHC, IP (not IF) |
| Lef-1 | Rabbit | C12A5 | 1:1000 | IF 1:100-1:400 | #2230 | Cell Signaling Technology | WB, IF, Flow (not IHC) |
| c-Myc | Rabbit | Polyclonal | 1:1000 | N/A | #9402 | Cell Signaling Technology | WB, IP, ChIP (not IF/IHC) |
| Cyclin D1 | Rabbit | 92G2 | 1:1000 | IHC validated | #2978 | Cell Signaling Technology | WB, IHC (not IF) |
| DKK2 | Rabbit | EPR23393-47 | 1:1000 | IHC 1:100-1:500 | ab278539 | Abcam | WB, IHC |
| Ki67 | Rabbit | SP6 | 1:100 | IF 1:1000, IHC 1:200 | ab16667 | Abcam | WB, IF, IHC, Flow |
| GAPDH | Rabbit | D16H11 | 1:1000 | IF validated, IHC validated | #5174 | Cell Signaling Technology | WB, IF, IHC |
| β-actin | Mouse | 8H10D10 | 1:1000 | IF validated, IHC validated | #3700 | Cell Signaling Technology | WB, IF, IHC, Flow |
| Anti-Rabbit IgG-HRP | Goat | Polyclonal | 1:2000 | - | #7074 | Cell Signaling Technology | WB |
| Anti-Mouse IgG-HRP | Horse | Polyclonal | 1:2000 | - | #7076 | Cell Signaling Technology | WB |
| Anti-Rabbit IgG-Alexa Fluor 488 | Goat | Polyclonal | - | 1:500 | A11008 | Invitrogen | IF, IHC |
| Anti-Rabbit IgG-Alexa Fluor 594 | Goat | Polyclonal | - | 1:500 | A11012 | Invitrogen | IF, IHC |

*Notes: WB, Western blot; IF, immunofluorescence; IHC, immunohistochemistry; IP, immunoprecipitation; Flow, flow cytometry; ChIP, chromatin immunoprecipitation; N/A, not applicable or not validated for this application;* CD81 (ab109201) is validated for WB only; not suitable for IF, IHC, or Flow Cytometry; CD200 (ab203887) is validated for IHC only; not suitable for WB or IF; Phospho-GSK3β (Ser9) (#9336) is validated for WB only; Anti-Mouse IgG-HRP (#7076) is Horse-sourced, not Goat-sourced; All Abcam antibodies listed are Rabbit-sourced (10 recombinant monoclonal, 2 polyclonal); β-actin (#3700) is Mouse-sourced; use #7076 (anti-Mouse) for detection.

**Table S2.** Primer Sequences for qRT-PCR

***mRNA Primers***

| **Gene** | **Forward Primer (5'→3')** | **Reverse Primer (5'→3')** | **Product Size (bp)** | **Accession No.** |
| --- | --- | --- | --- | --- |
| COL17A1 | AGCCAGTGAGACCCTGAATG | TGCTGGTAGGTCTCCTGGTT | 142 | NM_000494.4 |
| KRT15 | GAGGTGGAAGCCGAAGTATG | GTCTCCAGGTTCTGGATGGT | 156 | NM_002275.3 |
| CD200 | ATGGAGAGGCTGGTGATCTC | CTGCTGTCCTTGGTTCTGGT | 128 | NM_005944.6 |
| CTNNB1 | AAAATGGCAGTGCGTTTAG | TTTGAAGGCAGTCTGTCGTA | 135 | NM_001904.4 |
| LEF1 | AGAACACCCCGATGACGGA | GGCATCATTATGTACCCGGAAT | 148 | NM_016269.5 |
| CCND1 | GCTGCGAAGTGGAAACCATC | CCTCCTTCTGCACACATTTGAA | 135 | NM_053056.3 |
| MYC | GGCTCCTGGCAAAAGGTCA | CTGCGTAGTTGTGCTGATGT | 119 | NM_002467.6 |
| DKK2 | TGATGCGGAGACAAACAACG | TGTTCTTGGTTTCCATGTGCT | 143 | NM_014421.3 |
| AXIN2 | CAACACCAGGCGGAACGAA | GCCCAATAAGGAGTGTAAGGACT | 142 | NM_004655.4 |
| PTEN | TGGATTCGACTTAGACTTGACCT | GCGGTGTCATAATGTCTCTCAG | 126 | NM_000314.8 |
| PDCD4 | GTTGGCAGTATCCTTAGCATTGG | TCCACATCAGTTGTGCTCATCA | 131 | NM_014456.6 |
| GAPDH | GGAGCGAGATCCCTCCAAAAT | GGCTGTTGTCATACTTCTCATGG | 197 | NM_002046.7 |

***miRNA Primers***

| **miRNA** | **Forward Primer (5'→3')** | **Universal Reverse Primer** | **Accession No.** |
| --- | --- | --- | --- |
| hsa-miR-21-5p | GCCCGCTAGCTTATCAGACTGATG | mRQ 3' Primer (TaKaRa Kit) | MIMAT0000076 |
| hsa-miR-125b-5p | CGATTCTCCCTGAGACCCTAACT | mRQ 3' Primer (TaKaRa Kit) | MIMAT0000423 |
| hsa-let-7a-5p | GCGCTGAGGTAGTAGGTTGTATAG | mRQ 3' Primer (TaKaRa Kit) | MIMAT0000062 |
| hsa-miR-29a-3p | CGCGTAGCACCATCTGAAATC | mRQ 3' Primer (TaKaRa Kit) | MIMAT0000086 |
| U6 snRNA | CTCGCTTCGGCAGCACA | AACGCTTCACGAATTTGCGT | NR_004394.1 |

*Notes: All primers were synthesized by Sangon Biotech (Shanghai, China). qPCR conditions: 95°C for 30 s, followed by 40 cycles of 95°C for 5 s and 60°C for 30 s.*

**Table S3.** Complete List of miRNAs Detected in hUC-MSC Exosomes

| **Rank** | **miRNA** | **TPM** | **Percentage (%)** |
| --- | --- | --- | --- |
| 1 | hsa-miR-21-5p | 15,842 ± 2,136 | 12.67 |
| 2 | hsa-miR-125b-5p | 8,756 ± 1,245 | 7.00 |
| 3 | hsa-let-7a-5p | 6,423 ± 987 | 5.14 |
| 4 | hsa-miR-100-5p | 4,215 ± 634 | 3.37 |
| 5 | hsa-miR-29a-3p | 3,876 ± 521 | 3.10 |
| 6 | hsa-miR-23a-3p | 3,124 ± 478 | 2.50 |
| 7 | hsa-miR-221-3p | 2,856 ± 412 | 2.28 |
| 8 | hsa-miR-24-3p | 2,534 ± 389 | 2.03 |
| 9 | hsa-miR-199a-3p | 2,145 ± 324 | 1.72 |
| 10 | hsa-miR-126-3p | 1,987 ± 298 | 1.59 |
| 11 | hsa-let-7f-5p | 1,865 ± 276 | 1.49 |
| 12 | hsa-miR-27b-3p | 1,723 ± 258 | 1.38 |
| 13 | hsa-miR-143-3p | 1,584 ± 241 | 1.27 |
| 14 | hsa-miR-26a-5p | 1,456 ± 223 | 1.16 |
| 15 | hsa-let-7b-5p | 1,328 ± 205 | 1.06 |
| 16 | hsa-miR-222-3p | 1,215 ± 189 | 0.97 |
| 17 | hsa-miR-191-5p | 1,134 ± 176 | 0.91 |
| 18 | hsa-miR-27a-3p | 1,056 ± 165 | 0.84 |
| 19 | hsa-miR-30a-5p | 987 ± 154 | 0.79 |
| 20 | hsa-miR-145-5p | 923 ± 145 | 0.74 |
| 21 | hsa-miR-181a-5p | 865 ± 136 | 0.69 |
| 22 | hsa-miR-99b-5p | 812 ± 128 | 0.65 |
| 23 | hsa-miR-130a-3p | 764 ± 121 | 0.61 |
| 24 | hsa-miR-16-5p | 721 ± 115 | 0.58 |
| 25 | hsa-miR-10b-5p | 683 ± 109 | 0.55 |
| 26 | hsa-miR-152-3p | 648 ± 103 | 0.52 |
| 27 | hsa-miR-22-3p | 615 ± 98 | 0.49 |
| 28 | hsa-miR-146a-5p | 584 ± 93 | 0.47 |
| 29 | hsa-miR-103a-3p | 556 ± 89 | 0.44 |
| 30 | hsa-miR-148a-3p | 529 ± 85 | 0.42 |

*Notes: TPM, transcripts per million. Data represent mean ± SD from three independent exosome preparations.* *Raw read counts were not available from the sequencing service provider; only normalized TPM values are reported.*

**Table S4.** GO and KEGG Enrichment Analysis of miR-21-5p Target Genes

***GO Biological Process (Top 15)***

| **GO ID** | **Term** | **Gene Count** | **P-value** | **FDR** | **Representative Genes** |
| --- | --- | --- | --- | --- | --- |
| GO:0030178 | Negative regulation of Wnt signaling pathway | 28 | 2.3 × 10⁻⁸ | 1.8 × 10⁻⁵ | DKK2, SFRP1, WIF1, AXIN2 |
| GO:0007569 | Cell aging | 24 | 4.6 × 10⁻⁶ | 1.2 × 10⁻³ | CDKN1A, TP53, SIRT1, FOXO3 |
| GO:0043066 | Negative regulation of apoptotic process | 42 | 8.7 × 10⁻⁶ | 1.8 × 10⁻³ | BCL2, PTEN, PDCD4, SPRY1 |
| GO:0008285 | Negative regulation of cell proliferation | 38 | 1.2 × 10⁻⁵ | 2.1 × 10⁻³ | PTEN, CDKN1B, TIMP3, BTG2 |
| GO:0016055 | Wnt signaling pathway | 35 | 2.4 × 10⁻⁵ | 3.5 × 10⁻³ | DKK2, FZD4, LRP6, GSK3B |
| GO:0030308 | Negative regulation of cell growth | 22 | 3.8 × 10⁻⁵ | 4.8 × 10⁻³ | PTEN, PDCD4, RECK, TPM1 |
| GO:0001525 | Angiogenesis | 31 | 5.6 × 10⁻⁵ | 6.2 × 10⁻³ | VEGFA, HIF1A, SPRY1, TIMP3 |
| GO:0030512 | Negative regulation of TGF-β signaling | 18 | 7.8 × 10⁻⁵ | 7.6 × 10⁻³ | SMAD7, SKI, TGFBR2 |
| GO:0045786 | Negative regulation of cell cycle | 26 | 9.4 × 10⁻⁵ | 8.4 × 10⁻³ | CDKN1A, CDKN1B, RB1, TP53 |
| GO:0030335 | Positive regulation of cell migration | 29 | 1.2 × 10⁻⁴ | 9.8 × 10⁻³ | MMP2, MMP9, RECK, TIMP3 |
| GO:0006915 | Apoptotic process | 45 | 1.5 × 10⁻⁴ | 1.1 × 10⁻² | PDCD4, PTEN, BCL2, FASLG |
| GO:0008283 | Cell proliferation | 52 | 1.8 × 10⁻⁴ | 1.2 × 10⁻² | PTEN, CDKN1A, CCND1, MYC |
| GO:0001558 | Regulation of cell growth | 24 | 2.2 × 10⁻⁴ | 1.4 × 10⁻² | PTEN, IGF1, PDCD4, BTG2 |
| GO:0051591 | Response to cAMP | 15 | 2.8 × 10⁻⁴ | 1.6 × 10⁻² | CREB1, DUSP1, NR4A1 |
| GO:0045596 | Negative regulation of cell differentiation | 21 | 3.4 × 10⁻⁴ | 1.8 × 10⁻² | SOX2, NOTCH1, HES1, JAG1 |

***KEGG Pathway (Top 10)***

| **KEGG ID** | **Pathway** | **Gene Count** | **P-value** | **FDR** | **Representative Genes** |
| --- | --- | --- | --- | --- | --- |
| hsa04310 | Wnt signaling pathway | 32 | 1.8 × 10⁻⁷ | 2.4 × 10⁻⁵ | DKK2, SFRP1, FZD4, LRP6, GSK3B, AXIN2 |
| hsa04115 | p53 signaling pathway | 24 | 3.5 × 10⁻⁶ | 2.1 × 10⁻⁴ | TP53, CDKN1A, BCL2, PTEN, MDM2 |
| hsa04151 | PI3K-Akt signaling pathway | 48 | 8.2 × 10⁻⁶ | 3.6 × 10⁻⁴ | PTEN, AKT1, PIK3CA, FOXO3, BCL2 |
| hsa04110 | Cell cycle | 28 | 1.4 × 10⁻⁵ | 4.8 × 10⁻⁴ | CDKN1A, CDKN1B, CCND1, RB1, E2F1 |
| hsa04350 | TGF-β signaling pathway | 22 | 2.8 × 10⁻⁵ | 7.5 × 10⁻⁴ | SMAD7, TGFBR2, BMP4, BMPR2 |
| hsa04210 | Apoptosis | 26 | 4.5 × 10⁻⁵ | 1.0 × 10⁻³ | BCL2, PTEN, FASLG, CASP3, XIAP |
| hsa04060 | Cytokine-cytokine receptor interaction | 35 | 6.8 × 10⁻⁵ | 1.3 × 10⁻³ | IL6, IL1B, TGFB1, VEGFA |
| hsa04390 | Hippo signaling pathway | 24 | 8.9 × 10⁻⁵ | 1.5 × 10⁻³ | YAP1, TAZ, LATS1, MST1 |
| hsa05200 | Pathways in cancer | 56 | 1.2 × 10⁻⁴ | 1.8 × 10⁻³ | PTEN, TP53, MYC, VEGFA, BCL2 |
| hsa04550 | Signaling pathways regulating pluripotency | 21 | 1.8 × 10⁻⁴ | 2.2 × 10⁻³ | SOX2, NANOG, WNT3, FZD4 |

*Notes: FDR, false discovery rate. Analysis performed using clusterProfiler.*

**Table S5.** miR-21-5p Target Prediction Details for Wnt Pathway Components

| **Target Gene** | **Gene Full Name** | **3'UTR Length (bp)** | **Seed Match Type** | **Validation Status** | **Reference** |
| --- | --- | --- | --- | --- | --- |
| **DKK2** | Dickkopf WNT signaling pathway inhibitor 2 | 2,847 | 7mer-m8 | **Experimentally validated** | Kawakita et al., 2014 (PMID: 23999978) |
| PTEN | Phosphatase and tensin homolog | 3,812 | 8mer | Experimentally validated | Multiple studies |
| PDCD4 | Programmed cell death 4 | 2,156 | 8mer | Experimentally validated | Multiple studies |
| SPRY1 | Sprouty RTK signaling antagonist 1 | 1,987 | 7mer-m8 | Experimentally validated | Multiple studies |
| RECK | Reversion inducing cysteine rich protein with kazal motifs | 4,523 | 7mer-A1 | Experimentally validated | Multiple studies |
| TPM1 | Tropomyosin 1 | 1,654 | 7mer-m8 | Experimentally validated | Multiple studies |
| SMAD7 | SMAD family member 7 | 2,134 | 7mer-A1 | Experimentally validated | Multiple studies |

**miR-21-5p Binding Site in DKK2 3'UTR**

The interaction between miR-21-5p and DKK2 has been experimentally validated by Kawakita et al. (2014) through dual-luciferase reporter assay and Western blot analysis in oral cancer cells. miR-21 overexpression significantly reduced DKK2 protein levels, leading to Wnt/β-catenin pathway activation.

hsa-miR-21-5p seed sequence: 5'-AGCUUAU-3' (positions 2-8)

DKK2 3'UTR contains conserved miR-21-5p binding site

*Bold indicates the primary validated target in this study. Seed match types: 8mer (positions 2-8 match + position 1 A), 7mer-m8 (positions 2-8 match), 7mer-A1 (positions 2-7 match + position 1 A). PMID: PubMed identifier.*


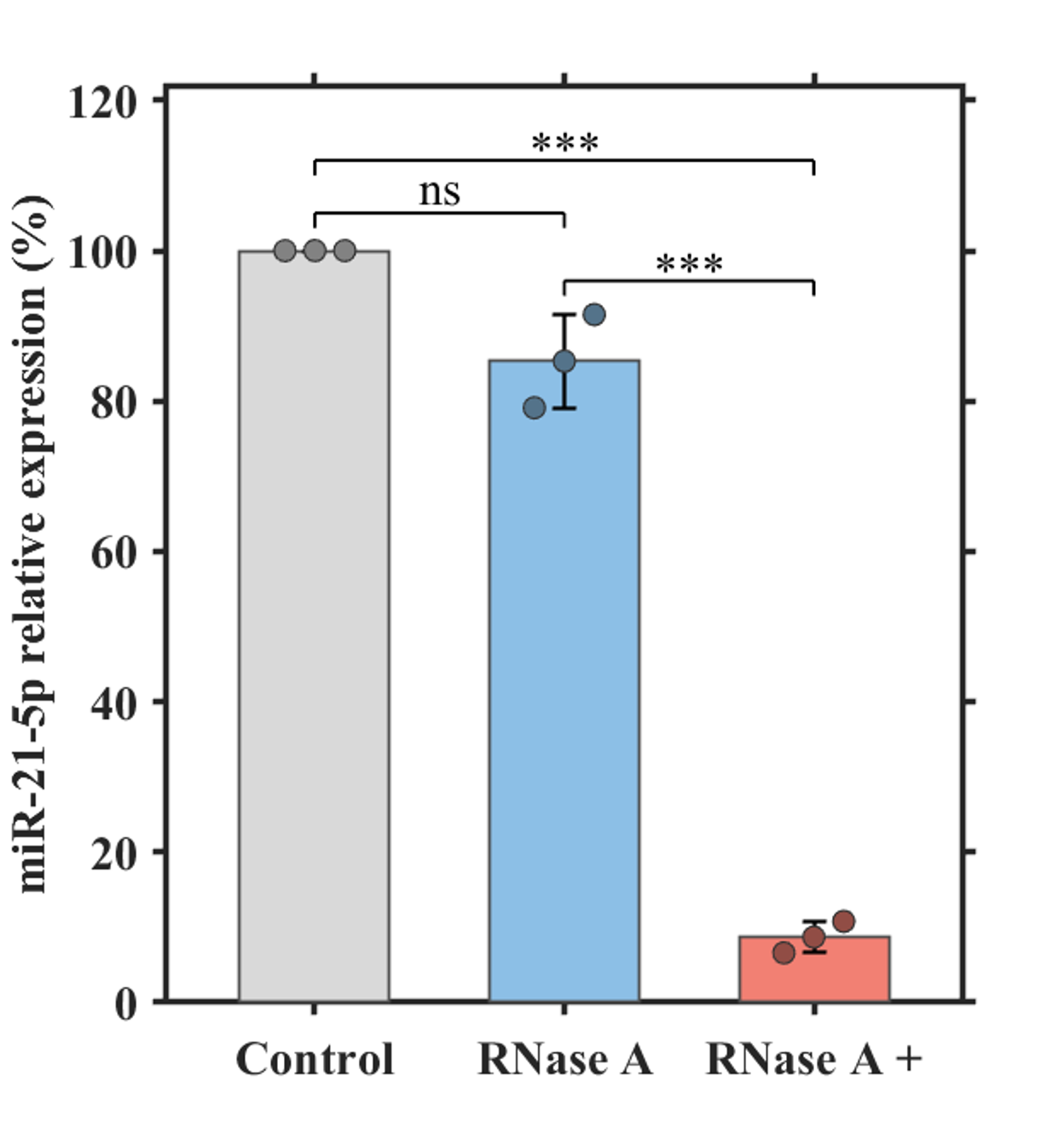


**Figure S1.** RNase A protection assay confirming intraluminal encapsulation of miR-21-5p in hUC-MSC-derived exosomes.

*
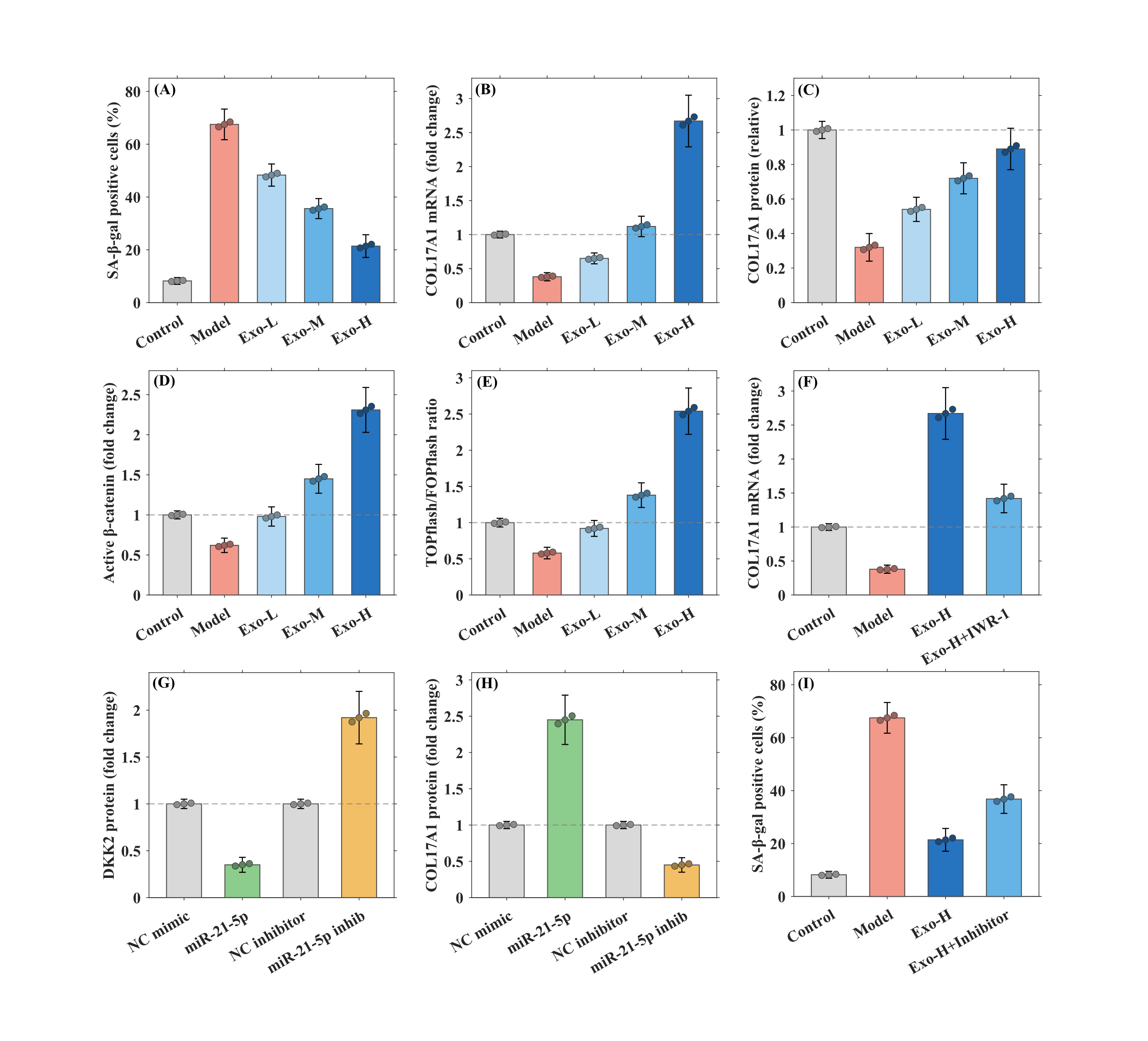
*

**Figure S2.** Raw data distribution for key quantitative endpoints. (A) SA-β-gal-positive cells (%) across dose groups (Figure 2); (B) COL17A1 mRNA expression (fold change) across dose groups (Figure 2); (C) COL17A1 protein levels (relative to control) across dose groups (Figure 2); (D) Active β-catenin levels (fold change) across dose groups (Figure 5); (E) TOPflash/FOPflash reporter activity across dose groups (Figure 5); (F) COL17A1 mRNA expression following IWR-1 Wnt pathway inhibition (Figure 5); (G) DKK2 protein levels following miR-21-5p mimic or inhibitor transfection (Figure 6); (H) COL17A1 protein levels following miR-21-5p mimic or inhibitor transfection (Figure 6); (I) SA-β-gal-positive cells (%) in miR-21-5p inhibitor rescue experiments (Figure 6).
